# Early upregulation of cytosolic phospholipase A_2_α in motor neurons is induced by misfolded SOD1 in a mouse model of amyotrophic lateral sclerosis

**DOI:** 10.1186/s12974-021-02326-5

**Published:** 2021-11-25

**Authors:** Yafa Fetfet Malada Edelstein, Yulia Solomonov, Nurit Hadad, Leenor Alfahel, Adrian Israelson, Rachel Levy

**Affiliations:** 1 Immunology and Infectious Diseases Laboratory, Department of Clinical Biochemistry and Pharmacology, Faculty of Health Sciences, Ben-Gurion University of the Negev and Soroka University Medical Center, 84105 Beer Sheva, Israel; 2 Department of Physiology and Cell Biology, Faculty of Health Sciences and The Zlotowski Center for Neuroscience, Ben-Gurion University of the Negev, Beer Sheva, Israel

**Keywords:** Cytosolic phospholipase A_2_α, ALS, Mutant SOD1^G93A^, Motor neurons, TNFα, Misfolded SOD1

## Abstract

**Background:**

Amyotrophic lateral sclerosis (ALS) is a fatal multifactorial neurodegenerative disease characterized by the selective death of motor neurons. Cytosolic phospholipase A_2_ alpha (cPLA_2_α) upregulation and activation in the spinal cord of ALS patients has been reported. We have previously shown that cPLA_2_α upregulation in the spinal cord of mutant SOD1 transgenic mice (SOD1^G93A^) was detected long before the development of the disease, and inhibition of cPLA_2_α upregulation delayed the disease’s onset. The aim of the present study was to determine the mechanism for cPLA_2_α upregulation.

**Methods:**

Immunofluorescence analysis and western blot analysis of misfolded SOD1, cPLA_2_α and inflammatory markers were performed in the spinal cord sections of SOD1^G93A^ transgenic mice and in primary motor neurons. Over expression of mutant SOD1 was performed by induction or transfection in primary motor neurons and in differentiated NSC34 motor neuron like cells.

**Results:**

Misfolded SOD1 was detected in the spinal cord of 3 weeks old mutant SOD1^G93A^ mice before cPLA_2_α upregulation. Elevated expression of both misfolded SOD1 and cPLA_2_α was specifically detected in the motor neurons at 6 weeks with a high correlation between them. Elevated TNFα levels were detected in the spinal cord lysates of 6 weeks old mutant SOD1^G93A^ mice. Elevated TNFα was specifically detected in the motor neurons and its expression was highly correlated with cPLA_2_α expression at 6 weeks. Induction of mutant SOD1 in primary motor neurons induced cPLA_2_α and TNFα upregulation. Over expression of mutant SOD1 in NSC34 cells caused cPLA_2_α upregulation which was prevented by antibodies against TNFα. The addition of TNFα to NSC34 cells caused cPLA_2_α upregulation in a dose dependent manner.

**Conclusions:**

Motor neurons expressing elevated cPLA_2_α and TNFα are in an inflammatory state as early as at 6 weeks old mutant SOD1^G93A^ mice long before the development of the disease. Accumulated misfolded SOD1 in the motor neurons induced cPLA_2_α upregulation via induction of TNFα.

**Supplementary Information:**

The online version contains supplementary material available at 10.1186/s12974-021-02326-5.

## Background

Amyotrophic lateral sclerosis (ALS) is a severe degenerative disorder, mainly affecting the motor neurons. Most of the cases (about 90%) are sporadic. Familial cases have been linked to mutations in several genes, including chromosome 9 open reading frame 72 (C9ORF72) repeat expansions, Cu/Zn superoxide dismutase (SOD1), TAR DNA binding protein (TDP-43) and others [1]. Mutant SOD1 is the best characterized form of familial ALS, accounting for 20% of familial cases [2]. It is generally believed that sporadic and familial ALS may share pathological mechanisms. The pathophysiology of the multifactorial-multisystemic ALS disease includes various mechanisms. Although ALS is not primarily considered an inflammatory or immune-mediated disease, inflammation appears to play a role in the pathogenesis of the disease in both ALS patients and animal models, inflammatory responses have been observed [3–5]. Microglia [6] and astrocytes [7] are activated during the progression of the disease, and evidence suggests that they contribute to neuronal death.

Previous findings suggested that cytosolic phospholipase A_2_α (cPLA_2_α) is regarded as an essential source of inflammation. cPLA_2_α specifically hydrolyzes phospholipids containing arachidonic acid at the sn-2 position [8, 9] and is the rate-limiting step in the generating eicosanoids and a platelet activating factor. These lipid mediators play critical roles in the initiation and modulation of inflammation and oxidative stress. cPLA_2_α is ubiquitous in all cells and is essential for their physiological regulation. However, elevated cPLA_2_α expression and activity were detected in the inflammatory sites in a vast array of inflammatory diseases, including neurodegenerative diseases [10–12]. Increased expression and activity of cPLA_2_α has been detected in neurons, astrocytes and microglia in the spinal cord, brainstem and cortex of sporadic ALS patients [13] and in the spinal cord of mutant SOD1^G93A^ transgenic mice [14], suggesting that cPLA_2_α may have an important role in the pathogenesis of the disease in all ALS patients. Our previous study [15] demonstrated that cPLA_2_α is upregulated in the spinal cord of 6 weeks old SOD1^G93A^ mice long before the appearance of the disease symptoms, neuronal death or gliosis, and remained elevated during the whole life span of the mice. Prevention of cPLA_2_α upregulation shortly before the onset of the disease symptoms, significantly delayed the loss of motor neuronal function, suggesting that cPLA_2_α upregulation in the spinal cord plays a role in the disease pathology. The mechanism that induces cPLA_2_α elevation in the spinal cord of as early as 6 weeks old mice, is not yet clear. SOD1 insoluble protein complexes (IPCs) were detected in motor neurons of 30 days old SOD1^G93A^ mice [16], before the manifestation of ALS pathology, and several months before the appearance of inclusion bodies. The present study aims to determine whether accumulated misfolded SOD1 triggers cPLA_2_α upregulation in motor neurons in the spinal cord of 6 weeks old ALS mice.

## Methods

### Animals

B6.Cg-Tg(SOD1G93A)1Gur/J hemizygous transgenic male mice were obtained from Jackson Laboratory (Bar Harbor, ME, U.S.A). The hemizygous transgenic male mice were also obtained by mating hemizygous transgenic males with C57BL/6J females (Jackson Laboratory). Each litter would generate hemizygous SOD1^G93A^ transgenic mice and littermate wild type controls as done before [15]. Transgenic male offspring were genotyped by PCR assay of DNA obtained from tail tissue (according to Jackson Laboratory). The study included male mice to avoid the estrogen effect. The study was approved by Ben-Gurion University Institutional Animal Care and Use Committee (IL-40-07-2016) and was conducted according to the Israeli Animal Welfare Act following the Guide for Care and Use of Laboratory Animal (National Research Council, 1996).

*Motor function measurement by Rotarod* A Rotarod test was used to evaluate the motor performance of the mice using an accelerating paradigm of 0.12 rpm/s as described before [15]. After a learning period of several days, mice were able to stay on the Rotarod (Rotamex-5, Columbus instruments, Columbus, OH, USA) for up to 150 s. Each mouse was given 3 trials and the best performance was used as a measure for motor function ability. Mice were tested twice a week from age of the 7 weeks-old until they could no longer perform the task.

*Spinal cord tissue preparation* Mice were deeply anesthetized and transcardially perfused with 20 ml of PBS [17].

*For immunoblot analysis or immunoprecipitation* Spinal cords were harvested in Lysis buffer containing 20 mM Tris pH7.5, 150 mM NaCl, 0.5% Sodium deoxycholate, 0.1% SDS, 0.1% Triton, 1 mM Phenylmethylsulfonyl fluoride and 1% protease inhibitors (Roche, Mannheim, Germany). The suspensions were sonicated 3 times for 20 s with Microsom Heatsystem Sonicator and centrifugated at 13,000×*g* for 20 min at 4 °C. Immunoprecipitation of cPLA_2_α or misfolded SOD1 was performed as described earlier [18, 19]. Spinal cords (100 µg) were solubilized in IP buffer (50 mM Tris–HCl pH 7.4, 150 mM NaCl, 1 mM EDTA, 0.5% Nonidet P-40, plus 1 × protease inhibitors) and incubated overnight with B8H10 antibodies (MédiMabs) or cPLA_2_α antibodies previously cross-linked to magnetic beads (Invitrogen, Waltham, Massachusetts, USA) with dimethyl pimelimidate (Pierce) according to the manufacturer’s instructions. The beads were magnetically isolated and washed three times with IP buffer. Samples were eluted by boiling in a 2 × SDS sample buffer. Lysate protein or resolved proteins were separated on 7% or 15% SDS-PAGE electrophoresis and transferred to nitrocellulose or PVDF membranes. Membranes were incubated in Tris-buffered saline (10 mM Tris, 135 mM NaCl, pH 7.4), with 0.1% Tween 20 (TBS-T) containing 5% non-fat milk for 1.5 h at 25 °C. The blots were then incubated with primary antibodies: 1:1000 rabbit anti-cPLA_2_ (Cell Signaling Danvers, MA USA), 1:250 mouse B8H10 anti-misfolded human SOD1 (Medimabs, Quebec, Canada), 1:1000 rabbit anti-calreticulin (Thermo Scientiific, IL, USA) as primary antibodies for overnight at 4 °C. After washing with TBS-T, they were incubated with secondary antibody: peroxidase conjugated goat anti-rabbit or anti mouse (Amersham Biosciences, Buckinghamshire, United Kingdom) for 1 h at 25 °C and developed using the enhanced chemiluminescence (ECL) detection system (PerkinElmer, Waltham, MA, USA). Proteins were quantified using video densitometry analysis (ImageJ version 4.0 Fuji).

*For immunostaining*—The spinal cords were fixed [15] in paraformaldehyde 4%/PBS solution overnight at 4 °C. The spinal cords were then transferred to PBS containing 30% sucrose for 24 h and then embedded in a 1:2 mixture of 30% sucrose in PBS:Tissue-Tek OCT (VWR, Radnor, PA), frozen in liquid nitrogen and stored at − 80 °C. Sections were made by cryostat (Leica Biosystems, Vienna, Austria) at 12 µm thickness, washed in PBS/tween 0.05%, incubated in PBS/Glycine 0.1% for 5 min and incubated in blocking solution (3% normal donkey serum and 2% BSA) at room temperature for 1 h. Then, these sections were incubated with primary antibodies diluted in blocking solution overnight at 4 °C. The primary antibodies used in the study were: 1:100 rabbit anti-cPLA_2_ (Santa Cruz Biotechnology, Santa Cruz, CA, USA), rabbit anti-pcPLA_2_α (Cell Signaling Danvers, MA USA), 1:100 mouse anti-misfolded human SOD1 (Medimabs, Quebec, Canada), 1:100 mouse anti-TNFα (Novus Biological USA, CO, USA), 1:100 goat anti-choline Acetyl-transferase (ChAT) (Millipore, CA, USA), 1:1000 rabbit anti-Iba-1 (Wako Pure Chemical Industries, Osaka, Japan), 1:500 rabbit anti-GFAP (Dako Glostrup Denmark), 1:100 mouse anti-GFAP (Millipore Darmstadt, Germany). Sections were washed with PBS/tween 0.05%, and incubated with 1:200 Cy3 or 1:100 anti-mouse Alexa488 or anti-rabbit Dylight conjugated secondary antibodies (Jackson Immunoresearch Laboratories, West Grove, PA, USA) for 1 h at room temperature. The staining of samples from the different treatments was performed in parallel. For each treatment, a negative control was prepared by omitting the primary antibody. Sections were mounted with anti-fading mounting medium (Electron Microscopy Sciences (EMS), Hatfield, PA, USA) and photographed in a blinded fashion using a fluorescent microscope (Olympus, BX60, Hamburg, Germany) or with confocal microscopy (Olympus, FluoView 1000, Tokyo, Japan). Using a confocal microscope, Z-sections were taken at 0.5 μm intervals and the results present Z-stack images. Fluorescence intensity was determined for cPLA_2_α using CellProfiler program. The % of fluorescence intensity of cell area was determined for the different cell types using CellProfiler program. LSM880 inverted laser-scanning confocal microscope (Zena, Germany) equipped with an Airyscan high-resolution detection unit and under identical acquisition conditions was also used. A Plain-Apochromat 63x/1.4 Oil DIC M27 objective was used, and parameters were set to avoid pixel intensity saturation and to ensure Nyquist sampling in the XY plane. Excitation of lasers for DAPI, Alexa 488 and Cy3 were 405 nm, 488 nm and 561 nm, respectively.

### Cell cultures 

**A** Motor neurons were isolated as described before with few modifications. Briefly, spinal cords were dissociated from C57BL/6J mouse embryos at day 13.5 (E13.5) with 2 mg/ml papain for 25 min at 37 °C, and triturated with 0.5% bovine serum albumin (BSA) and 0.01 mg/ml DNase I in Leibovitz’s L-15 medium (Gibco). Cells were then triturated with Leibovitz’s L-15 media and the single-cells suspension was separated through Optiprep gradient (Sigma) and seeded at a density of 50,000 cells/ 24 well on coverslips pre-covered polyornithine (Sigma) and laminin (Sigma). The motor neurons were cultured with Neurobasal media (Gibco) supplemented with B27 (Gibco), 2% horse serum (Sigma), 1 ng/ml CNTF (R&D systems), and 1 ng/ml GDNF (R&D systems) and maintained at 37 °C in a 5% CO_2_ humidified incubator. At 6 DIV, glial cells were inhibited using 5-Fluoro-2′-deoxyuridine (Sigma) and uridine (Sigma). For expression of human SOD1 in motor neurons, the cells were infected at 7 DIV using AAV1/2-SOD1^WT^ or AAV1/2-SOD1^G93A^ as previously described [20].

**B** Motor neuron like NSC34 cell line—were maintained in Dulbecco’s modified Eagle’s medium (DMEM) supplemented with 10% fetal calf serum (FCS) and 1% penicillin/streptomycin solution at 37 °C with 5% CO_2_ and were sub-cultured every 2–3 days [21]. For differentiation, the proliferation medium (DMEM, 10% FCS, 1% P/S) was exchanged 24 h after seeding with a differentiation medium containing 1:1 DMEM/F-12 (Ham), 1% FCS, 1% modified Eagle’s medium nonessential amino acids (NEAA), 1% P/S and 1 μM all- trans retinoic acid. SOD1^WT^ and SOD1^G93A^ were constructed and purified as described before [22]. Transfection was performed by using TurboFect (Thermo) according to the manufacturer’s protocol. TNF-α-neutralizing antibody (Cell Signaling Technology, Danvers, MA, USA) was used to study its effect.

*Cell lysates*—were prepared using lysis buffer containing: 2% Triton X-100, 50 mM HEPES (pH 7.5), 150 mM NaCl, 1 mM EDTA, 1 mM EGTA, 10% glycerol, 10 μm MgCl_2_, 10 μg/ml leupeptin, 1 mM phenyl-methylsulphonylfluoride, 10 μg/ml aprotonin, 1 mM benzamidine, 20 mM para-nitrophenyl phosphate, 5 mM sodium orthovanadate, 10 mM sodium fluoride, and 50 mM β-glycerophosphate). Cell lysates were analyzed by SDS-PAGE on 15–10% gels. The amount of protein in each sample was quantified with the Pierce BCA Proteins Assay using BSA standards. The resolved proteins were transferred to PVDF membrane and blocked in 5% BSA in TBS-T (10 mM Tris, 135 mM NaCl, pH 7.4, 0.1% Tween 20). The detection of immunoreactive bands was carried out as described above but for cPLA_2_α, using 1:500 rabbit anti cPLA_2_α (GeneTex Inc, Alton Pkwy Irvine, CA, USA).

*TNF*α* levels*—were measured by a TNFα high sensitivity ELISA, eBioscience, Vienna, Austria.

### Statistical analysis

Data were expressed as mean ± standard error of the mean (SEM). Statistical significance was determined by either one- or two-way analysis of variance (ANOVA) followed by a posteriori Bonferroni’s test for multiple comparisons provided by GraphPad Prism version 5.00 for Windows (GraphPad Software, San Diego, CA, USA). Pearson coefficient correlation (*r*) was used to study the relationships between the variables.

## Results

**Fig. 1:**
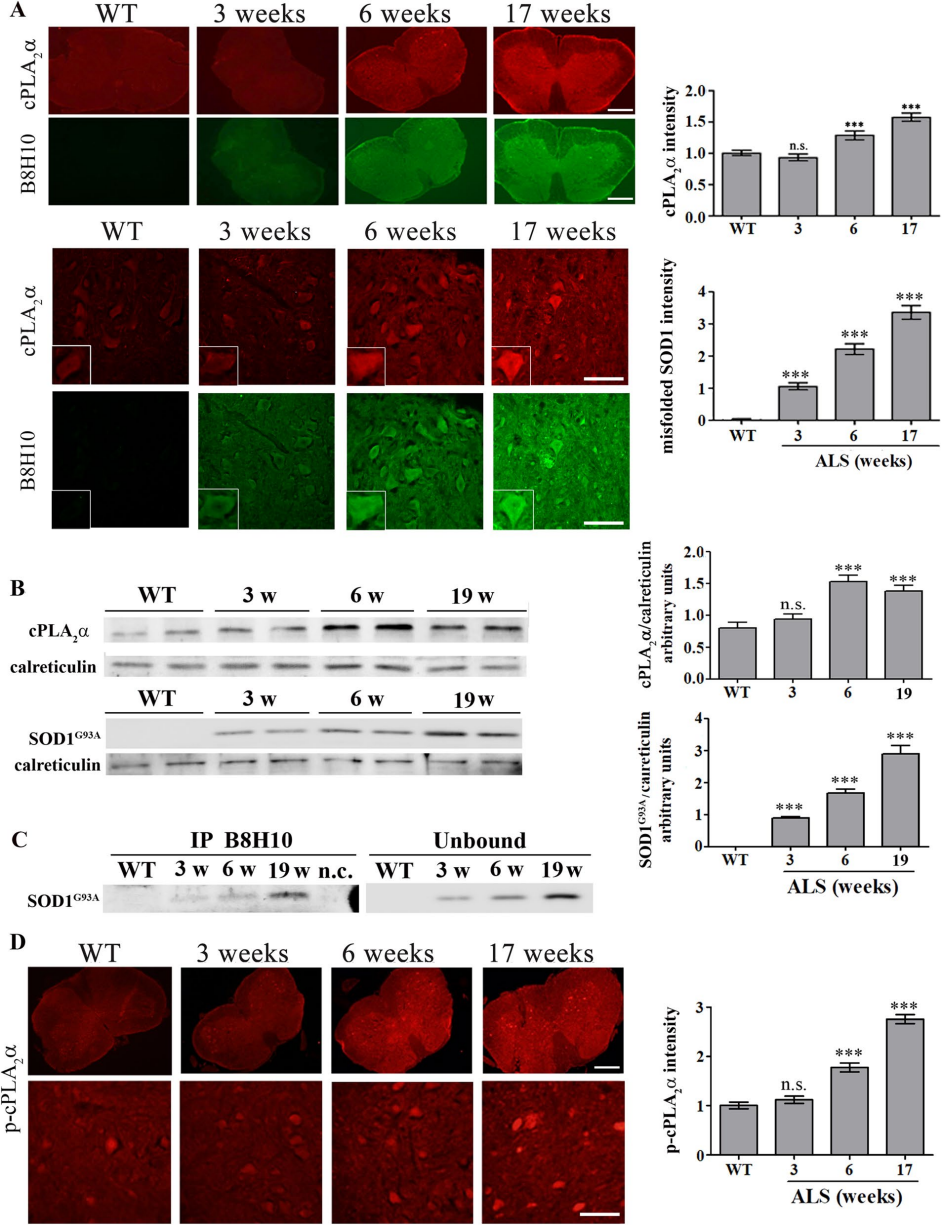
Misfolded SOD1 accumulation precedes cPLA_2_α upregulation and activation. **A** A representative double immunofluorescence staining of cPLA_2_α (red) and misfolded SOD1 (B8H10, green) proteins in the lumbar spinal cord sections of WT and mutant SOD1^G93A^ mice during the development of the disease (3, 6 and 17 weeks). Scale bars = 500 μm (upper panel), 100 μm (lower panel) and inset 20 μm. The means ± SEM fluorescence intensity for both magnifications are presented in the bar graph as arbitrary units. Four mice for each time point and five fields for each mouse were analyzed. ***p < 0.001 compared to control mice (WT). n.s. non-significant. **B** A representative immunoblot analysis of cPLA_2_α, mutant SOD1^G93A^ and their corresponding calreticulin protein expression in the spinal cord lysates of WT and mutant SOD1^G93A^ mice during the development of the disease. cPLA_2_α protein expression was determined by dividing the intensity of each cPLA_2_α or mutant SOD1^G93A^ (SOD1^G93A^) by the intensity of the corresponding calreticulin band after quantitation by densitometry and expressed in the bar graph as arbitrary units. The bar graphs are the means ± SE of 4 mice in each group. ***p < 0.001 significance—compared to control mice (WT). n.s. non-significant. **C**. Misfolded SOD1 was immunoprecipitated using anti-B8H10 antibodies from spinal cord lysates of WT and mutant SOD1^G93A^ mice during the development of the disease. A representative immunoblot analysis of the misfolded SOD1 (IP B8H10, left) and 10% of the unbound (Unbound, right) fractions are shown. nc- negative control, immunoprecipitation without anti-B8H10 antibodies. **D** A representative immunofluorescence staining of phosphor-cPLA_2_α (p-cPLA_2_α) in the lumbar spinal cord sections of WT and mutant SOD1^G93A^ mice during the development of the disease (3, 6 and 17 weeks). Scale bars = 500 μm (upper panel) and 100 μm (lower panel). The means ± SEM fluorescence intensity for both magnifications are presented in the bar graph in arbitrary units. Four mice for each time point and five fields for each mouse were analyzed. ***p < 0.001 significance compared to WT mice. n.s. non-significant

In our previous study we reported that cPLA_2_α is elevated in the spinal cord of 6 weeks old mutant SOD1^G93A^ mice but not at 3 weeks. To study whether cPLA_2_α is affected by the accumulation of misfolded SOD1 in the cells, cPLA_2_α and misfolded SOD1 proteins expression and accumulation were analyzed in the spinal cord of SOD1^G93A^ mice. Immunofluorescence staining and quantitation showed a significant (*p* < 0.001) elevation of cPLA_2_α protein expression in the spinal cord sections (Fig. 1A) of 6 weeks old SOD1^G93A^ mice, as shown in our previous study [15]. Immunofluorescence staining and quantitation of misfolded SOD1 showed that it was significantly (*p* < 0.001) detected in the spinal cord at 3 weeks old SOD1^G93A^ mice, before the elevation of cPLA_2_α. The expression of cPLA_2_α and mutant SOD1^G93A^ was also determined by western blot analysis and showed that mutant SOD1^G93A^ was detected at 3 weeks preceding the elevation of cPLA_2_α (Fig. 1B). Moreover, misfolded SOD1 determined by immunoprecipitation with anti B8H10 was detected at 3 weeks in the spinal cord of SOD1^G93A^ mice and gradually increased at a later age (Fig. 1C). Activation of cPLA_2_α analyzed by immunostaining of phosphor- cPLA_2_α was detected at the spinal cord section of 6 weeks old SOD1^G93A^ mice but not at 3 weeks (Fig. 1D).

**Fig. 2:**
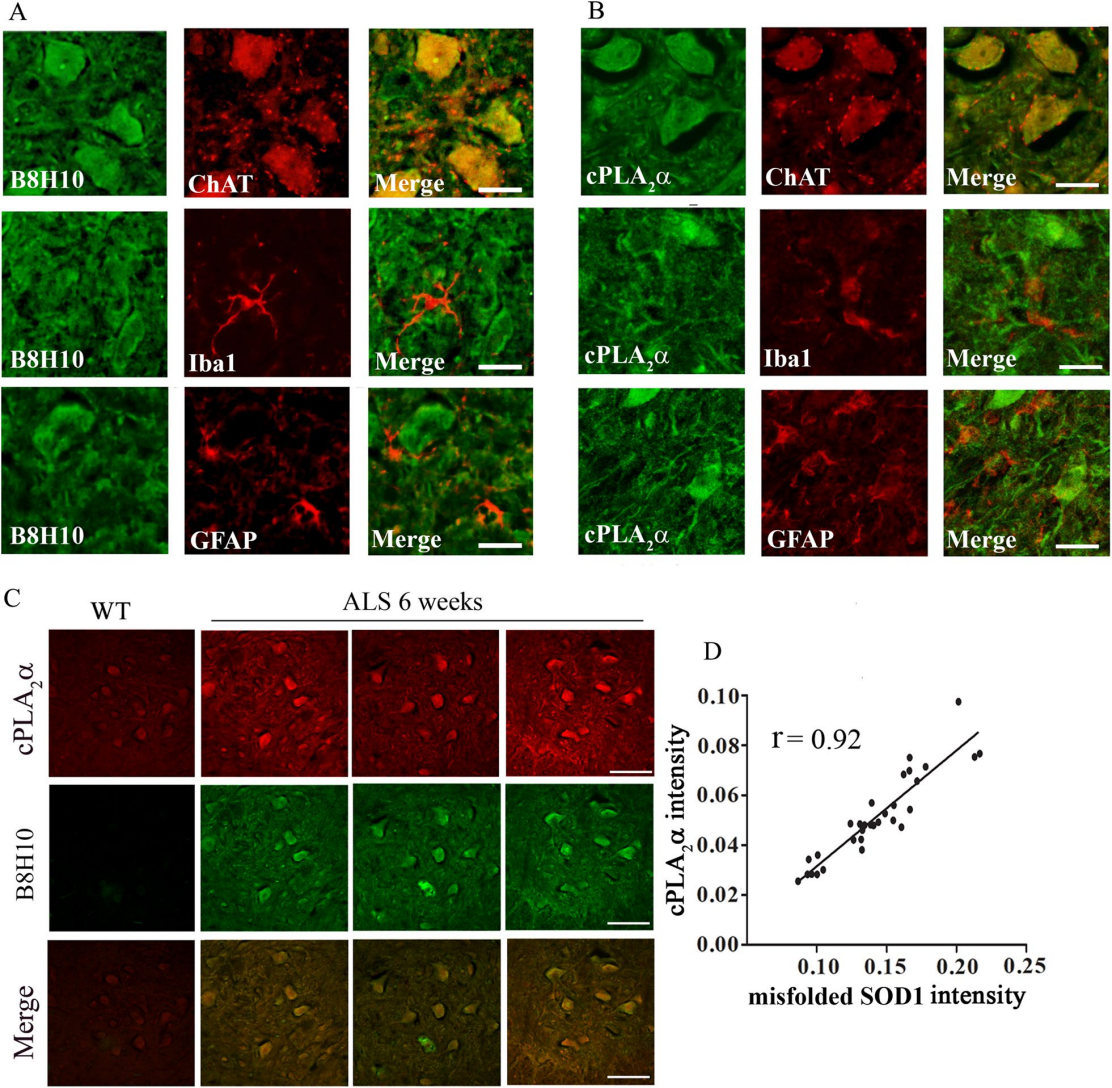
cPLA_2_α and misfolded SOD1 accumulates specifically in motor neurons of young pre-symptomatic SOD1^G93A^ mice. **A** Double staining (red) of motor neurons (ChAT), microglia (Iba-1) or astrocytes (GFAP) and misfolded SOD1 (B8H10, green) in the spinal cord sections of 6 weeks old pre-symptomatic SOD1^G93A^ mice. Scale bars = 20 μm. Shown a representative out of five mice. **B** Double staining (red) of motor neurons (ChAT), microglia (Iba-1) or astrocytes (GFAP) and cPLA_2_α (green) in the spinal cord sections of 6 weeks old pre-symptomatic SOD1^G93A^ mice. Scale bars = 20 μm. Shown a representative out of five mice. **C** Double immunofluorescence staining of cPLA_2_α (red) with misfolded SOD1 (B8H10, green) proteins in the spinal cord sections of control mice (WT) and 6 weeks old pre-symptomatic mutant SOD1^G93A^ mice. Scale bars = 100 μm. **D** The Pearson coefficient correlation (r) between cPLA_2_α and misfolded SOD1 in the motor neurons of the intensity of cPLA_2_α and misfolded SOD1 expressed in arbitrary units of immunostaining as presented in the representative results in C. Four filed in each of the 8 different mice analyzed

**Fig. 3:**
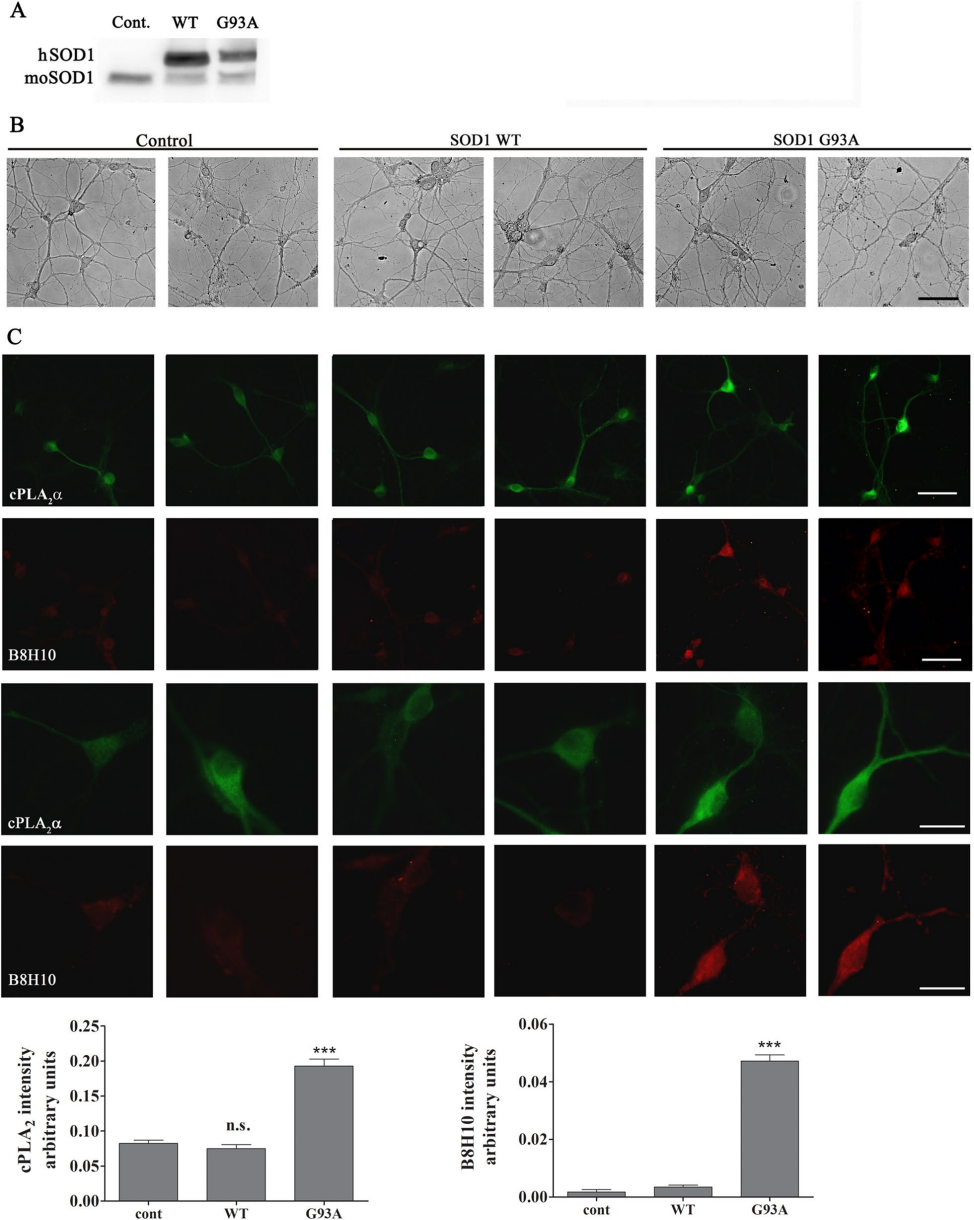
Accumulation of misfolded SOD1 in primary motor neurons induced cPLA_2_α upregulation. **A** Immunoblot analysis of human SOD1^WT^ or mutant SOD1^G93A^ (hSOD1), expressed in primary motor neurons by infection of AAV1/2 as described in materials and methods. Immunoblot analysis of mouse SOD1 (mSOD1) is shown as a control. **B** Light microscope pictures of primary motor neurons. Control—without infection, motor neurons expressing human wild type SOD1 (SOD1 WT) or mutant SOD1 (SOD1 G93A). Scale bars = 50 μm. **C** Double immunofluorescence staining of cPLA_2_α (green) with misfolded SOD1 (B8H10, red) in motor neurons expressing SOD1^WT^, mutant SOD1^G93A^ and control cells. Two upper panels scale bars = 50 μm and two lower panels scale bars = 20 μm. 3 different independent experiments were analyzed and showed similar results. The means ± SEM fluorescence intensity for cPLA_2_α and misfolded SOD1 is presented in the bar graphs as arbitrary units. Five fields in each of the 3 different treatments of motor neurons in each experiment was analyzed. Significance compared to control ***p < 0.001, n.s. non-significant

The elevated accumulation of misfolded SOD1 and the elevated cPLA_2_α protein expression in the spinal cord sections of 6 weeks old mutant SOD1^G93A^ mice were detected specifically in motor neurons as determined by co-immunofluorescence staining of misfolded SOD1 and the marker of motor neurons ChAT (Fig. 2A). Using specific antibodies against misfolded SOD1 showed that elevated misfolded SOD1 was already detected at 3 weeks (Additional file 1: Fig. S1), and co-staining with ChAT showed that it is expressed in the motor neurons (Additional file 2: Fig. S2). Co-immunofluorescence staining of misfolded SOD1 and Iba1 or GFAP, the markers of microglia or astrocytes, respectively, showed that misfolded SOD1 is not accumulated in these cells at 6 weeks (Fig. 2A). As shown by co-immunofluorescence staining, elevated cPLA_2_α expression was also detected specifically in the motor neurons and not in microglia or astrocytes (Fig. 2B). Co-immunostaining of cPLA_2_α and misfolded SOD1 showed co-localization and overlapping between cPLA_2_α and misfolded SOD1 in the motor neurons in the spinal cord of 6 weeks old mutant SOD1^G93A^ mice (Fig. 2C). cPLA_2_α upregulation showed some variation that highly correlated with the accumulation of misfolded SOD1 with a correlation coefficient of 0.92 between both proteins, suggesting that the level of misfolded SOD1 in the motor neurons defines the level of cPLA_2_α protein expression (Fig. 2D). To determine whether the accumulation of misfolded SOD1 in the motor neurons is responsible for cPLA_2_α upregulation, human SOD1^WT^ or mutant SOD1^G93A^ were expressed in primary motor neurons isolated from the spinal cord from C57BL/6J mouse embryos as shown in the western blot analysis (Fig. 3A). The expression of human SOD1 did not affect the morphology of the motor neurons and their number (Fig. 3B). Double staining with anti -B8H10 and anti-cPLA_2_α showed elevated cPLA_2_α protein expression in motor neurons expressing mutant SOD1^G93A^ and accumulating misfolded SOD1 (Fig. 3C). In motor neurons that did not accumulate misfolded SOD1 (as shown in Fig. 3A), cPLA_2_α expression did not change and was similar to that detected in control motor neurons (without induction). These results clearly indicate that misfolded SOD1 induced cPLA_2_α upregulation.

**Fig. 4:**
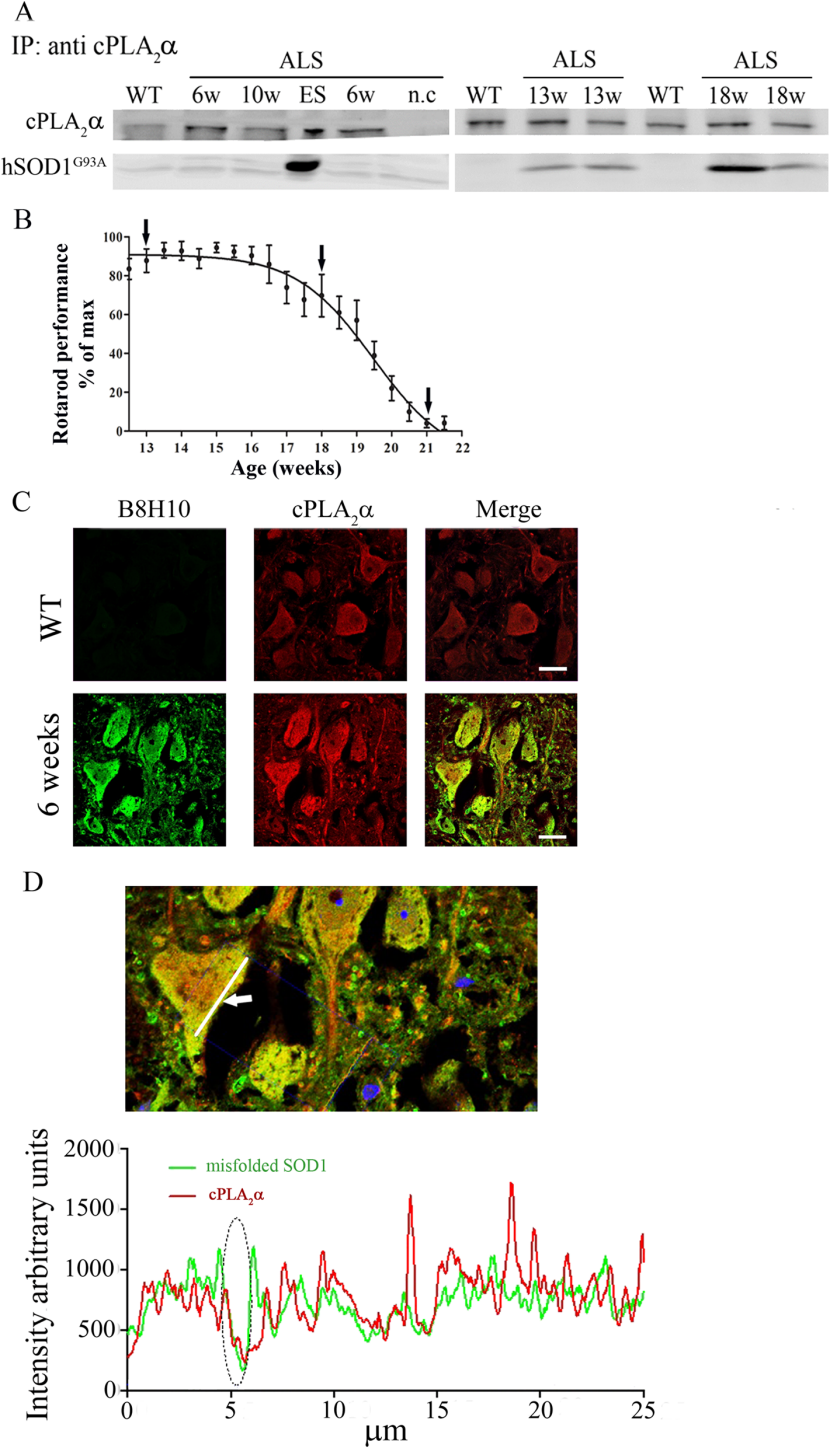
Partial binding between cPLA_2_α and mutant SOD1^G93A^ in motor neurons of 6 weeks old mutant SOD1^G93A^ mice. **A** Immunoprecipitation with antibody against cPLA_2_α and Western Blot analysis for cPLA_2_α and mutant SOD1 in the spinal cord lysate of control (WT) and mutant SOD1^G93A^ mice during the development of the disease shown by a representative immunoblot. Negative control (n.c). -without antibodies against cPLA_2_α. **B** Motor performance on accelerating Rotarod test (*n* = 10 mice). The arrows show the immunoprecipitation analysis along the development of the disease. **C** Airyscan detector high resolution confocal microscopy images of double immunofluorescence staining of cPLA_2_α (red) with misfolded SOD1 (B8H10, green) proteins in the spinal cord sections of 6 weeks old pre-symptomatic SOD1^G93A^ mouse. Scale bars = 20 μm. **D** The coupling of both proteins (cPLA_2_α and misfolded SOD1) in the line shown by the arrow was analyzed and presented as intensity along the line. The ellipse shows an example of binding between both proteins

To study whether the elevated cPLA_2_α protein expression is triggered or stabilized by an interaction between misfolded SOD1 and cPLA_2_α, the binding between both proteins was determined by co-immunoprecipitation experiments. As shown in Fig. 4A, immunoprecipitation of cPLA_2_α in the spinal cord lysates at disease onset (13 weeks), symptomatic stage (18 weeks) or end stage (Fig. 4B) resulted in a significant co-immunoprecipitation of mutant SOD1^G93A^ (Fig. 4A), suggesting a binding between them. In contrast, there was no co-immunoprecipitation of mutant SOD1^G93A^ and cPLA_2_α in the spinal cord lysates of 6 weeks old mutant SOD1^G93A^ mice, suggesting that there is no binding between the proteins in that early stage. We then used an Airyscan detector, a sub-diffraction high-resolution laser-scanning confocal microscope to examine the binding between both proteins. The Airyscan detector used in the current study provides improved lateral resolution (~ 150 nm) and signal to noise ratio, as compared with conventional confocal microscopes. Under these conditions, accurate and straight forward analysis of the interaction between misfolded SOD1 and cPLA_2_α in the motor neurons in the spinal cord section was allowed. Airyscan high-resolution detection showed that there is only partial overlapping indicating partial binding between both proteins at 6 weeks (Fig. 4C, D).

**Fig. 5:**
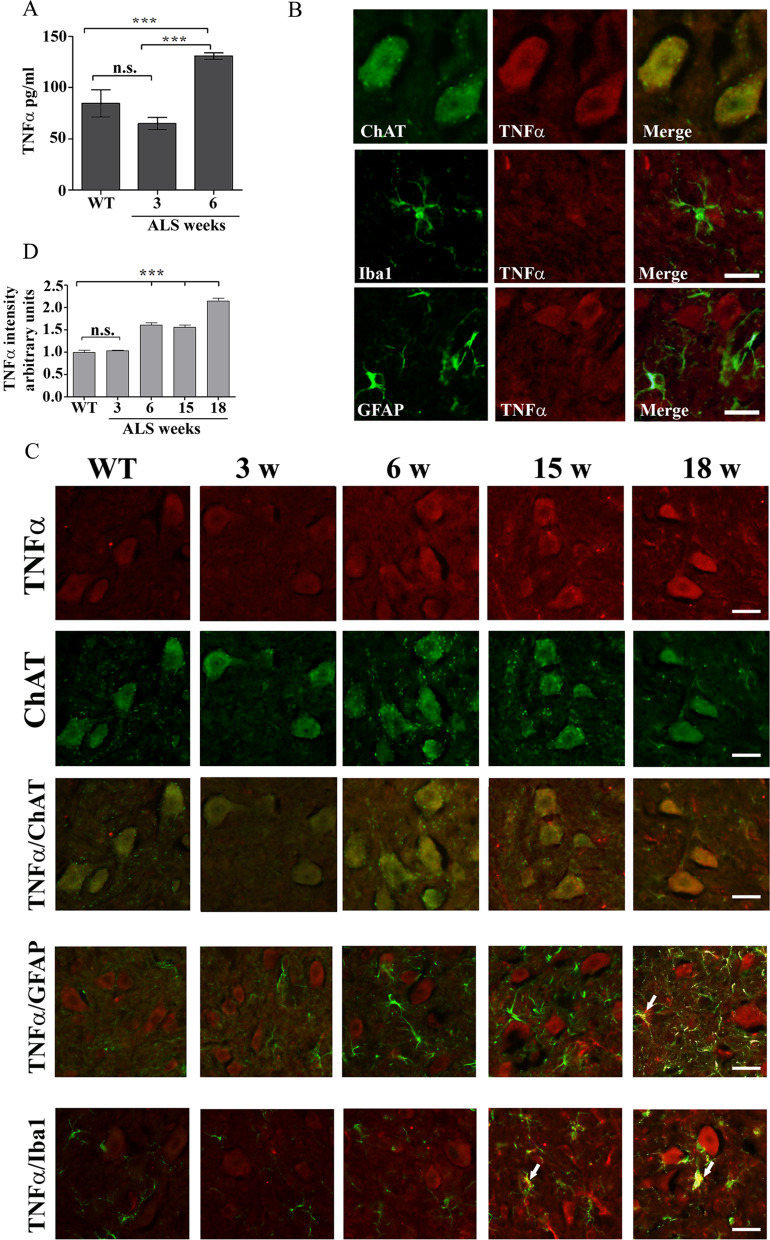
Increased TNFα is restricted to the motor neurons of pre-symptomatic 6 weeks old mutant SOD1^G93A^ mice. **A** The levels of TNFα in the spinal cord lysate of WT and of 3 and 6 weeks old pre-symptomatic mutant SOD1^G93A^ mice detected by ELISA. Significance—***p < 0.001, n.s. = non-significant. The bar graph is the mean ± SE of 8 mice in each group. **B** Representative double immunofluorescence staining cell markers (green) of motor neurons (ChAT), microglia (Iba-1) or astrocytes (GFAP) and TNFα (red) in the spinal cord sections of 6 weeks old pre-symptomatic SOD1^G93A^ mice. Scale bars = 20 μm. 3 other mice in each group were analyzed and showed similar results. **C** A representative time course of double immunofluorescence staining of TNFα (red) and cell markers (green) of motor neurons (ChAT), microglia (Iba-1) or astrocytes (GFAP) proteins in the lumbar spinal cord sections of WT and mutant SOD1^G93A^ mice during the course of the disease (3, 6, 15 and 18 weeks). Scale bars = 20 μm. 3 other mice in each group were analyzed and showed similar results. D. The means ± SEM fluorescence intensity for TNFα is presented in the bar graph as arbitrary units. Four mice for each time point and five fields for each mouse were analyzed. Significance compared to control ***p < 0.001, n.s. non-significant

**Fig. 6:**
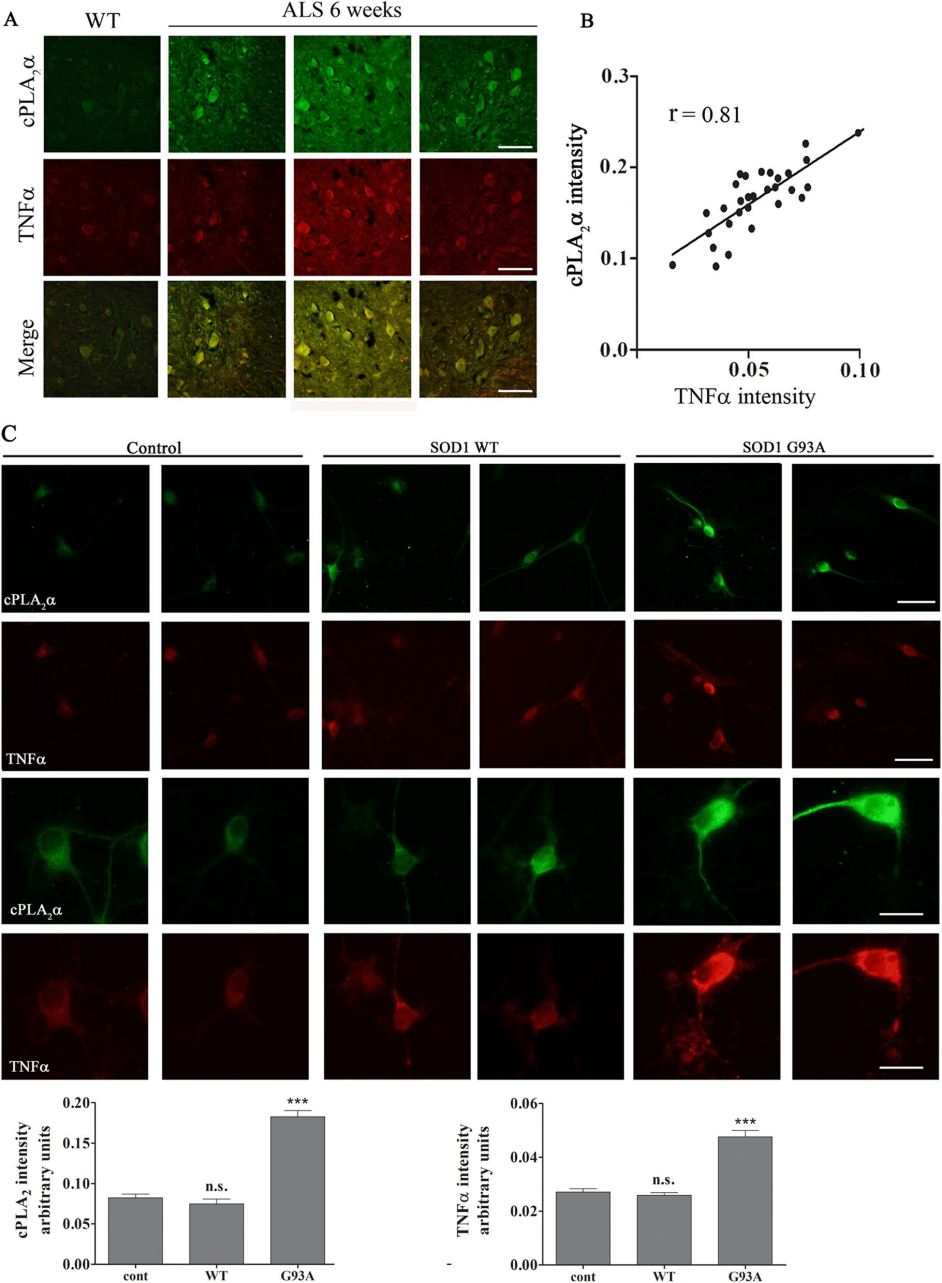
Elevated cPLA_2_α protein expression in the motor neurons is highly correlated with TNFα. **A** Double immunofluorescence staining of cPLA_2_α (green) and TNFα (red) proteins in the lumbar spinal cord sections of WT and 6 weeks old mutant SOD1^G93A^ mice. Scale bars = 100 μm. **B** The Pearson coefficient correlation (r) between cPLA_2_α and TNFα in the spinal motor neurons of mutant SOD1^G93A^ mice was analyzed. Florescence intensity is expressed in arbitrary units of immunostaining as presented in the representative results in A. Four fields in each of the 8 different mice analyzed. **C** Elevated cPLA_2_α and TNFα in primary motor neurons expressing mutant SOD1^G93A^. Double immunofluorescence staining of cPLA_2_α (green) and TNFα (red) in primary motor neurons expressing human SOD1^WT^, mutant SOD1^G93A^ and control cells described in Fig. 3. Two upper panels, scale bars = 50 μm and two lower panels, scale bar s = 20 μm. 3 different independent experiments were analyzed and showed similar results. The means ± SEM fluorescence intensity for cPLA_2_α and TNFα is presented in the bar graphs as arbitrary units. Five fields in each of the 3 different treatments of motor neurons in each experiment was analyzed. Significance compared to control ***p < 0.001, n.s. non-significant

**Fig. 7:**
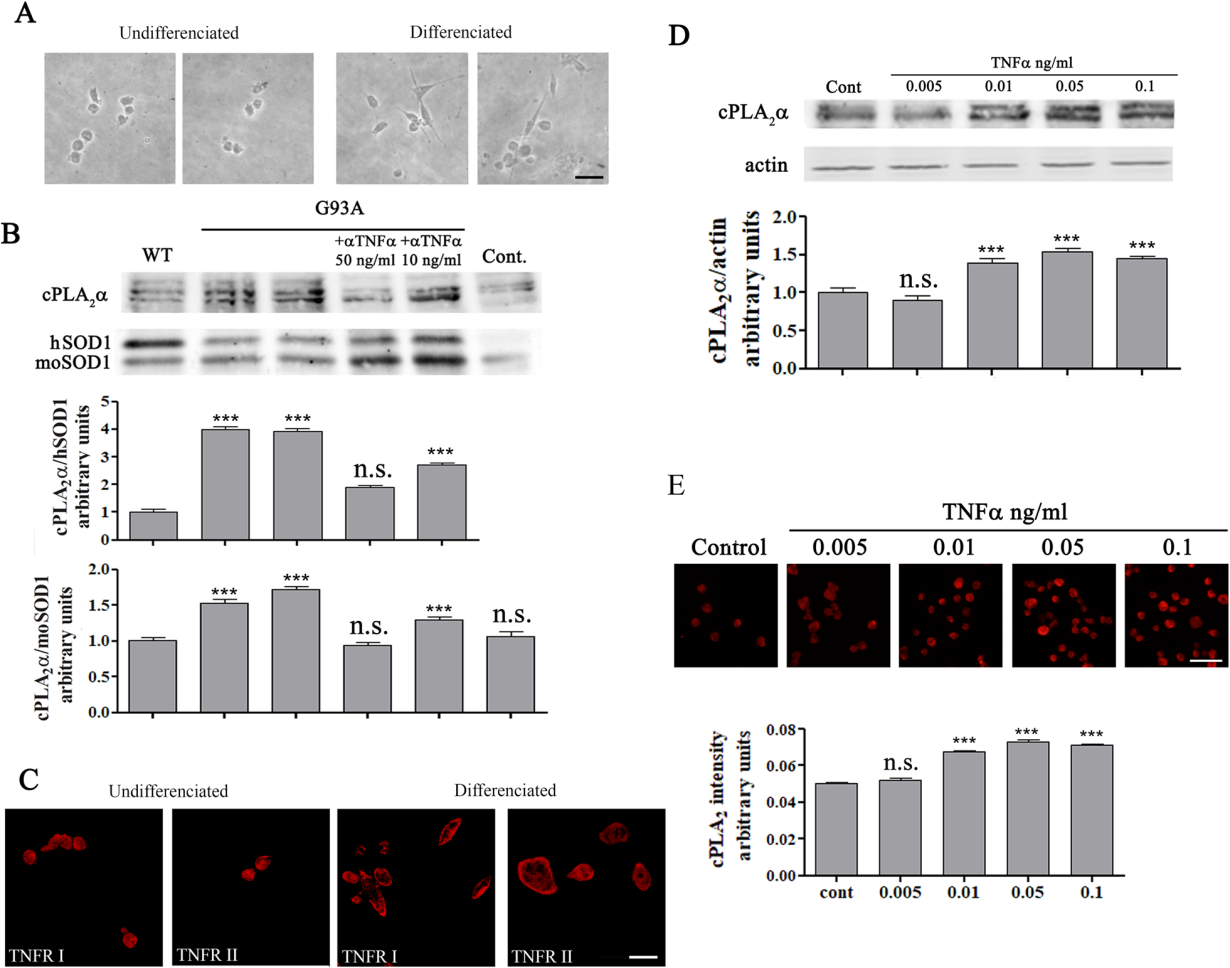
Secreted TNFα is responsible for cPLA_2_α upregulation. **A** Representative light microscope pictures of undifferentiated and differentiated motor like NSC34 cells, scale bars = 50 μm. **B** cPLA_2_α upregulation induced by mutant SOD1^G93A^ expressing cells is prevented in the presence of anti-TNFα antibodies. NSC-34 cells were transfected with human SOD1^WT^ or mutant SOD1^G93A^ plasmids for 72 h before lysates preparations. TNFα neutralizing antibodies were added at 48 h. A representative immunoblot analysis of cPLA_2_α in NSC34 cells transfected with human SOD1^WT^ (WT) or mutant SOD1^G93A^ (G93A) in the absence or presence of anti-TNFα. Control cells without transfection (Cont.). cPLA_2_α protein expression was determined by dividing the intensity of each cPLA_2_α with the intensity of the corresponding human SOD1 or mouse SOD1 after quantitation by densitometry and expressed in the bar graph as arbitrary units. The bar graphs are the means ± SE of three experiments. Significance compared to control ***p < 0.001, n.s. non-significant. **C** Representative confocal pictures of immunofluorescence staining of TNFRI and TNFRII in undifferentiated and differentiated motor like NSC34 cells, scale bars = 20 μm. Two other experiments showed similar results. **D** A representative immunoblot analysis of a dose dependent effect of TNFα (0.005–0.1 ng/ml) for 24 h on cPLA_2_α expression in differentiated motor neuron-like NSC34 cells. cPLA_2_α protein expression was determined by dividing the intensity of each cPLA_2_α with the intensity of the corresponding actin after quantitation by densitometry and expressed in the bar graph as arbitrary units. The bar graphs are the means ± SE of 3 experiments. Significance compared to control ***p < 0.001, n.s. non-significant. **E** A representative immunofluorescence staining of a dose dependent effect of TNFα (0.05–0.1 ng/ml) for 24 h on cPLA_2_α expression in differentiated motor neuron-like NSC34 cells. Scale bars = 50 μm. The bar graph is the mean ± SE of 3 different experiments. Significance compared to control ***p < 0.001, n.s. non-significant

Proinflammatory cytokines such as TNFα [23, 24] are shown to induce elevation of cPLA_2_α protein expression, thus, we examined whether increased levels of TNFα could be detected in the spinal cord of 6 weeks old mutant SOD1^G93A^ mice. As shown in Fig. 5A, there is a significant (*p* < 0.001) elevation of TNFα in the spinal cord lysates of 6 weeks old mutant SOD1^G93A^ mice in comparison with spinal cord lysates of WT or 3 weeks old SOD1^G93A^ mice (130.0 ± 3.0 pg/ml compared with 85.5 ± 13.3 and 73.6 ± 6.7 pg/ml, respectively). To determine which type of cell produces TNFα, co-immunofluorescence staining of TNFα using anti-TNFα antibodies that show specific staining (Additional file 3: Fig. S3) and the different cell markers was performed in the spinal cord sections of 6 weeks old SOD1^G93A^ mice. Co-immunofluorescence staining of TNFα and ChAT clearly showed that TNFα was detected in the motor neurons in the spinal cords of 6 weeks old mutant SOD1^G93A^ mice (Fig. 5B). Immunofluorescence staining of the motor neurons in the spinal cord of 6 weeks old SOD1^G93A^ mice showed elevation of both TNFα receptors expression in comparison to WT mice (Additional file 4: Fig. S4). Co- immunofluorescence staining of TNFα and either Iba1 or GFAP showed that TNFα was not detected in the microglia or astrocytes at 6 weeks (Fig. 5B). A time course of co-immunofluorescence staining of TNFα and the different cell markers in the spinal cords of SOD1^G93A^ mice clearly shows elevated TNFα levels in the motor neurons at 6 weeks which was gradually increased at a later stage (Fig. 5C). TNFα was not detected in glia cells at 6 weeks, but was detected in microglia at 15 weeks and in astrocytes at 18 weeks (Fig. 5C). In accordance with these results, immunofluorescence staining of Iba1 or GFAP, to determine glia activation, showed no significant activation in the spinal cord section of mutant SOD1^G93A^ mice at these early stages (3 and 6 weeks), although misfolded SOD1 was already accumulated in the spinal cord, but did show a significant activation in the spinal cord sections of 17 weeks old SOD1^G93A^ mice (Additional file 5: Fig. S5) as reported in our previous study [15]. Co-immunofluorescence staining and densitometry analysis of cPLA_2_α and TNFα in the spinal cord sections of 6 weeks old SOD1^G93A^ mice showed as above that there is a variation of elevated cPLA_2_α protein expression in the different mice (Fig. 6A), which was highly correlated with TNFα (coefficient correlation, *r* = 0.81) in the motor neurons (Fig. 6B), suggesting that TNFα produced in the motor neurons is responsible for cPLA_2_α upregulation. We next studied the effect of misfolded SOD1 accumulation on TNFα expression using motor neurons that were viral induced with human SOD1^WT^ or mutant SOD1^G93A^ (described in Fig. 3). As shown in Fig. 6C, double staining of cPLA_2_α and TNFα showed that motor neurons with accumulated mutant SOD1^G93A^ (Fig. 3C) expressed elevated levels of both cPLA_2_α and TNFα. Over expression of SOD1^WT^ did not affect cPLA_2_α and TNFα compared with the control cells. The comparison between motor neurons expressing SOD1^WT^ and those expressing the mutant SOD1^G93A^ clearly shows that of the accumulation of misfolded SOD1 induces the elevation of cPLA_2_α and TNFα (Additional file 6: Fig. S6). To determine whether elevated TNFα is responsible for cPLA_2_α upregulation, human SOD1^WT^ or SOD1^G93A^ were expressed in the motor neurons like NSC34 cells. NSC34 is an hybrid cell line produced by the fusion of motor neurons from the spinal cords of mouse embryos with mouse neuroblastoma cells N18TG2 that exhibit properties of motor neurons after differentiation [21] as presented by shape change (Fig. 7A). As clearly shown in the western blot analysis (Fig. 7B), accumulation of mutant SOD1 caused significant elevation of cPLA_2_α, while accumulation of SOD1^WT^ did not affect on cPLA_2_α expression in NSC34 cells compared with the control (untransfected cells). The presence of neutralizing TNFα antibodies (50 ng/ml) prevented cPLA_2_α upregulation, indicating that TNFα is responsible for cPLA_2_α upregulation by an autocrine mechanism. In accord with these results, although very low levels of TNFα were detected in the supernatant of the cells, they were significantly higher in supernatant of cells transfected with human SOD1^G93A^ compared with cells transfected with SOD1^WT^ or control cells (Additional file 7: Fig. S7). The differentiated neuron motors NSC34 cells express both TNF receptors as shown by immunofluoresnce staining (Fig. 7C). The addition of TNFα to differentiated NSC34 cells caused cPLA_2_α upregulation in a dose dependent manner, as shown by western blot analysis (Fig. 7D) and immunofluorescence staining cPLA_2_α (Fig. 7E).

## Discussion

The present study clearly demonstrates that misfolded SOD1 is significantly detected in the spinal cords of 3 weeks old mutant SOD1^G93A^ mice preceding the elevated expression of cPLA_2_α and its activation at 6 weeks. The elevation of cPLA_2_α in the spinal cords at 6 weeks, was detected specifically in motor neurons and not in microglia or astrocytes. While, at the symptomatic stage, elevated cPLA_2_α was also detected in the glia cells in accordance with our and others previous studies [14, 15]. Similar to our results, activation and elevation of cPLA_2_α protein expression were mainly detected in motor neurons in other pathological conditions such as after spinal cord injury and in spinal inflammatory hyperalgesia [25–29]. cPLA_2_α is a major inflammatory enzyme-producing arachidonic acid, a substrate for the formation of eicosanoids and a platelet-activating factor which are well-known mediators of inflammation and tissue damage implicated in pathological states of several acute and chronic neurological disorders [26, 30–32]. The detection of elevated and activated cPLA_2_α in the motor neurons at 6 weeks old mutant SOD1^G93A^ mice and long before any neuronal damage or sign of the disease is evident, indicates an inflammatory state of the motor neurons at this very early stage. Misfolded SOD1 accumulation in the spinal cord of 3 weeks old SOD1^G93A^ mice was detected in motor neurons but not in astrocytes or microglia. The significant accumulation of misfolded SOD1 in the motor neurons before the appearance of elevated cPLA_2_α expression and the significant correlation (*r* = 0.92) between both proteins at 6 weeks shown in the present study raised the possibility that the accumulation of misfolded SOD1 in the cells dictates the expression of cPLA_2_α. Indeed, the accumulation of misfolded SOD1 (determined by B8H10 staining) in primary motor neurons isolated from the mouse spinal cord or expression of mutant SOD1 in NSC34 motor neuron-like cells caused a significant elevation of cPLA_2_α protein expression. In contrast, expression of human SOD1^WT^ did not affect cPLA_2_α expression, indicating that intracellular misfolded SOD1 induced cPLA_2_α upregulation. Although the role of glial cells in neuronal damage and disease progression is well established [33], we show here that the elevation of cPLA_2_α in the motor neurons at 6 weeks is independent of glia cells and occurs long before any neuronal damage. In accordance with our results, the effect of accumulated mutant SOD1 on cPLA_2_α in NSC34 cells was reported recently [34]. They showed that expression of SOD1^G93A^ in motor neuron cell line NSC34 for long time induced cell death mediated by cPLA_2_α. The elevated levels of cPLA_2_α in familial and sporadic ALS [13] together with the observations that inclusions containing misfolded SOD1 are regularly present in motor neurons of ALS patients, both with and without SOD1 mutations [35], support the notion that misfolded SOD1 accumulated in the motor neurons contributes to the elevated cPLA_2_α expression.

Induction or stabilization of proteins by other proteins within the cells via an interaction mechanism such as induction of P53 by elevated amyloid beta [36] and stabilization of EGFR or Kit C by HIP1 binding have been reported [37, 38]. In addition, binding and co-precipitation of oligomeric or misfolded SOD1 with other proteins including voltage dependent anion channel (VDAC1) [39, 40], macrophage migration inhibitory factor (MIF) [22, 41], heat shock protein [42], glutathione peroxidase 1 [43] or Bcl-2 [44] have been documented. Taken together, these observations and the results demonstrating high overlapping of misfolded SOD1 and cPLA_2_α in the motor neurons in spinal cord sections by confocal microscopy could suggest that cPLA_2_α upregulation is induced by its interaction with misfolded SOD1 at 6 weeks old mutant SOD1^G93A^ mice. Co-immunoprecipitation of both proteins was detected only at the symptomatic stage but not at 6 weeks, indicating no direct interaction at this stage. Using the Airyscan detector, a sub-diffraction high-resolution laser-scanning confocal microscope [45, 46], we showed that only partial binding between both proteins in motor neurons at 6 weeks, explains the absence of co-immunoprecipitation at this stage, and questioning the possibility that the interaction between misfolded SOD1 and cPLA_2_α induced the elevation of cPLA_2_α expression.

cPLA_2_α was shown to be induced by different proinflammatory mediators and insults through specific receptors or scavenger receptors [23, 47–50]. Ours and other studies reported that TNFα induced cPLA_2_α upregulation in various systems [23, 51, 52] and in motor neuron-like NSC34 cells, as demonstrated in the present study. In the neural environment, constitutive physiological levels of TNFα regulate synaptic plasticity, modulates dendritic maturation, pruning, and synaptic connectivity to respond to alterations in sensory stimuli to maintain homeostatic plasticity [53, 54]. Overexpression of TNFα has been associated with neuronal excitotoxicity, synapse loss, and propagation of the inflammatory state [55]. TNFα elicits its wide range of biological responses by activating two distinct receptors, TNF-R1 and TNF-R2 [56–58]. Antigenic TNFα and its soluble receptors measured by ELISA were significantly higher in ALS patients than in healthy controls [59]. To our knowledge, the present study is the first to show a significant elevation of TNFα protein in the spinal cord of mutant SOD1^G93A^ mice as early as at 6 weeks, that was about 150% of the levels of TNFα in the spinal cord of WT mice and of 3 weeks old mutant SOD1^G93A^ mice. Our results are supported by other studies reporting that TNFα was detected in the spinal cord of late pre-symptomatic stage ALS mice. TNFα was the sole cytokine whose mRNA could be observed in the spinal cord of young pre-symptomatic SOD1^G93A^ mice [60]. A microarray survey of 1081 gene products expressed in spinal cords of SOD1^G93A^ mice reported that TNFα was the only inflammatory cytokine found to be differentially expressed [4]. Upregulation of TNFα and its proapoptotic receptors mRNA were detected at late pre-symptomatic stages and preceded transcriptional upregulation of other pro-inflammatory gene products and temporally correlates with the progression of the disease in SOD1^G93A^ mice [61, 62]. TNFα was reported to be elevated in the spinal cord of SOD1^G93A^ transgenic mice in the early life span, at 80 days [63]. We also show here for the first time that elevated TNFα is expressed specifically in spinal motor neurons of 6 weeks old SOD1^G93A^ mice but not in microglia or astrocytes, although glia cells are reported to be the major cell type to secrete TNFα [64]. In agreement with our results, immunohistochemical analysis showed little TNFα immunoreactivity in motor neurons from 60 days old SOD1^G93A^ transgenic mice with a healthy appearance [65] and FasL as early as day 40 [65]. Since FasL is upregulated by TNFα [66], it is possible that due to the methodology sensitivity TNFα was not detected in the spinal cord of 40 days old SOD1^G93A^ mice but at 60 days in their study [65]. We show here a high correlation (*r* = 0.81) between cPLA_2_α and TNFα expressed in the spinal motor neurons of 6 weeks old SOD1^G93A^ mice, suggesting that misfolded SOD1 induced the elevation of cPLA_2_α via production of TNFα. Indeed, as we show in the present study, that expression of mutant SOD1 but not SOD1^WT^ in motor neurons induced both cPLA_2_α and TNFα upregulation. Moreover, the presence of neutralizing TNFα antibodies prevented the elevation of cPLA_2_α expression NSC34 like motor neurons, indicating that TNFα is responsible for cPLA_2_α upregulation, acting via its autocrine effect. Likewise, addition of TNFα (10–100 pg/ml) to differentiated NSC34 cells for 24 h caused cPLA_2_α upregulation in a dose dependent manner similar to the concentration detected in the spinal cord of 6 weeks of SOD1^G93A^ mice. In agreement with our report, addition of soluble TNFα (acting through a reverse signaling) for 6 days affected motor neurons, inducing a marked motor neuron loss in SOD1-G93A monocultures [33]. Since elevated TNFα receptors were detected in motor neurons in the spinal cord of 6 weeks old SOD1^G93A^ mice, in accordance with others that reported elevated TNFR in the pre-symptomatic stage [67], the elevated TNFα in the spinal cord at this time point probably acts through its receptors to induce cPLA_2_α upregulation.

Our results, suggesting that motor neurons have a crucial role in inflammatory state (demonstrating elevated both cPLA_2_α and TNFα) during the early stage of the disease, are in accordance with the specific activation of motor neurons but not glia cells in the pre-symptomatic stage (at 8 weeks) of the disease in mutant SOD1^G93A^ mice evident by the increased p38MAPK [67, 68], activation of ASK1, MKK3,4,6, overexpression of both TNFα receptors (TNFR1 and TNFR2) [67] and TNFα accumulation in transgenic motor neurons [33]. In addition, motor neurons were reported as a primary determinant of disease onset and early disease progression by selective mutant gene inactivation within the cells [69]. Moreover, it was shown [70] that neuronal expression of mutant SOD1 was sufficient to cause motor neuron degeneration and paralysis in transgenic mice with cytosolic dendritic ubiquitinated SOD1 aggregates as the dominant pathological feature. Crossing neuron-specific mutant SOD1 mice with ubiquitously wild-type SOD1-expressing mice led to dramatic wild-type SOD1 aggregation in oligodendroglia after the onset of neuronal degeneration suggesting that mutant SOD1 in neurons triggers neuronal degeneration, which in turn may facilitate aggregates formation in surrounding glial cells. In contrast, cell-specific deletion of mutant SOD1 in genetically altered mice has implicated microglia and astrocytes as contributors to the late disease progression but not the onset of disease [71–73].

## Conclusions

We show here that elevated protein expression of both cPLA_2_α and TNFα were detected specifically in motor neurons and not in glial cells in the spinal cord of 6 weeks old SOD1^G93A^ mice, indicating the inflammatory state of the motor neurons long before the development of signs of the disease. Misfolded SOD1 is accumulated in the spinal cord motor neurons of 3 weeks old SOD1^G93A^ mice, preceding cPLA_2_α and TNFα upregulation. The results of the present study show: **a**. The high correlation between cPLA_2_α and misfolded SOD1 levels and between cPLA_2_α and TNFα levels in the motor neurons at 6 weeks, **b**. cPLA_2_α and TNFα upregulation by expressing mutant SOD1 in primary motor neurons and in NSC34 motor neurons like cells, **c**. the prevention of cPLA_2_α upregulation in the presence of TNFα neutralizing antibodies and **d**. the induction of cPLA_2_α upregulation by addition of TNFα and the presence of TNFR receptors. Based on these results we can conclude that accumulated misfolded SOD1, in the motor neurons in the spinal cord of 6 weeks old SOD1^G93A^ mice, induced cPLA_2_α upregulation mediated by TNFα via its autocrine effect.

## Supplementary Information

**Additional file 1: Figure S1.** Specificity of immunofluorescence analysis of misfolded SOD1. Representative results of immunofluorescence analysis of misfolded SOD1, using B8H10 antibodies, versus negative control (n.c.) and wild type (WT) in mice spinal cords. Scale bar = 100 μm.

**Additional file 2: Figure S2.** Misfolded SOD1 could be detected at 3 weeks in motor neurons. A representative double immunofluorescence of motor neurons marker (ChAT) and misfolded SOD1 in the spinal cord of 3 weeks old SOD1^G93A^ mice. Scale bar = 100 μm

**Additional file 3: Figure S3.** Specificity of immunofluorescence analysis of TNFα. Representative results of immunofluorescence staining of TNFα versus negative control (n.c.) in the spinal cord of WT mice and 6 weeks old SOD1^G93A^ mice. Scale bar = 100 μm. There is a low staining of TNFα in the spinal cord of WT mice in accordance with the low level of TNFα in their lysates.

**Additional file 4: Figure S4.** TNFα receptors are elevated in motor neurons in the spinal cord of 6 weeks old mutant SOD1^G93A^ mice. Representative immunofluorescence staining of TNFRI and TNFRII in the spinal cord of WT mice and mutant SOD1^G93A^ mice. Scale bars = 50 μm. The elevated receptors are detected in motor neurons as determined by the cell shape.

**Additional file 5: Figure S5.** The accumulation of misfolded SOD1 precedes glia activation. Representative immunofluorescence staining of Iba1, GFAP (red) or misfolded SOD1 (B8H10, green) proteins in the lumbar spinal cord sections of WT and mutant SOD1^G93A^ mice during the course of the disease (3, 6 and 17 weeks). Scale bars = 100 μm. The mean ± SEM fluorescence intensity expressed by arbitrary units is presented in the bar graph (*n* = 4 mice for each time point, five fields were analyzed for each mouse). ***p < 0.001—compared to control mice (WT). n.s. = non significant.

**Additional file 6: Figure S6.** Detection of cPLA_2_α and TNFα in motor neurons expressing hSOD1^WT^ or mutant hSOD1^G93A^. The Pearson coefficient correlation between cPLA_2_α and misfolded SOD1 (r = 0.87) and between cPLA_2_α and TNFα (r = 0.96) in motor neurons expressing mutant SOD1^G93A^. Representative results of florescence intensity of immunostaining presented in Figs. 3C and 6C is expressed in arbitrary units.

**Additional file 7: Figure S7.** Elevated TNFα levels in supernatant of NSC34 cells expressing SOD1^G93A^. The levels of TNFα in the supernatants of NSC34 cells transfected with human SOD1^WT^ or mutant SOD1^G93A^ plasmids (as described in Fig. 6B) detected by ELISA. The bar graph is the mean ± SE of three experiments. Significance compared to control ***p < 0.001, n.s. non-significant.

## Data Availability

There is no data, software, databases, and application/tool available apart from the reported in the present study. All data generated or analyzed during this study are included in this published article are provided in the manuscript and its Additional files.

## Abbreviations

ALS: Amyotrophic lateral sclerosis
cPLA2α: Cytosolic phospholipase A2 alpha
SOD1^G93A^: SOD1 transgenic mice
TNFα: Tumor necrosis factor alpha
hSOD1^G93A^: Recombinant human mutant SOD1^G93A^
WT: Wild type
