# Supplementary figures and images for: Early upregulation of cytosolic phospholipase A2α in motor neurons is induced by misfolded SOD1 in a mouse model of amyotrophic lateral sclerosis
Source: J Neuroinflammation. 2021 Nov 25;18:274. doi: 10.1186/s12974-021-02326-5 (PMC8620709; doi:10.1186/s12974-021-02326-5)

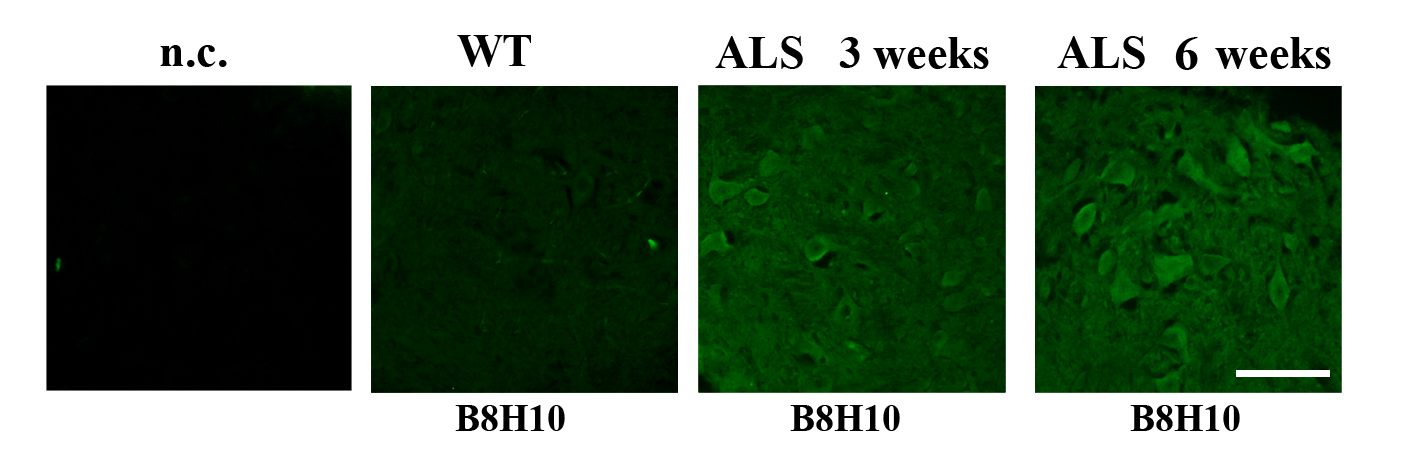

Supplement: Supplementary file 1 — Additional file 1: Figure S1. Specificity of immunofluorescence analysis of misfolded SOD1. Representative results of immunofluorescence analysis of misfolded SOD1, using B8H10 antibodies, versus negative control (n.c.) and wild type (WT) in mice spinal cords. Scale bar = 100 μm. [file 12974_2021_2326_MOESM1_ESM.tif]

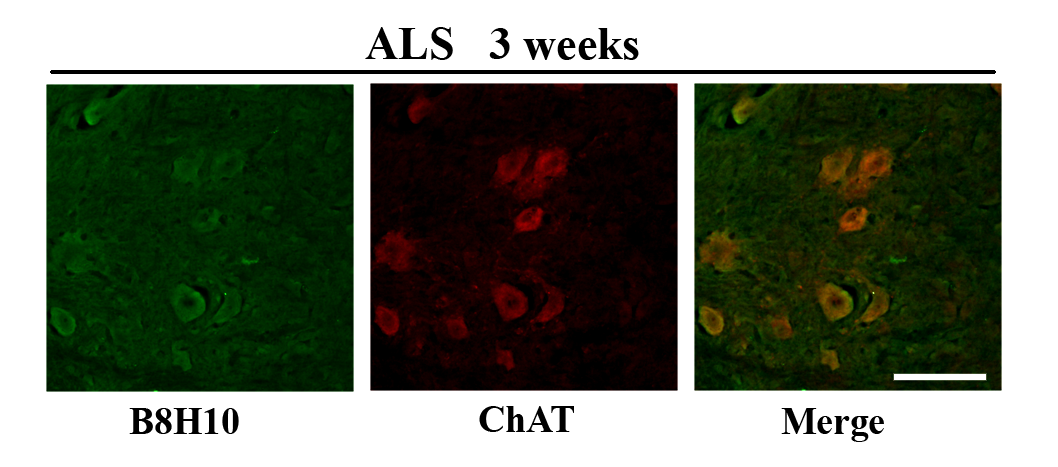

Supplement: Supplementary file 2 — Additional file 2: Figure S2. Misfolded SOD1 could be detected at 3 weeks in motor neurons. A representative double immunofluorescence of motor neurons marker (ChAT) and misfolded SOD1 in the spinal cord of 3 weeks old SOD1G93A mice. Scale bar = 100 μm [file 12974_2021_2326_MOESM2_ESM.tif]

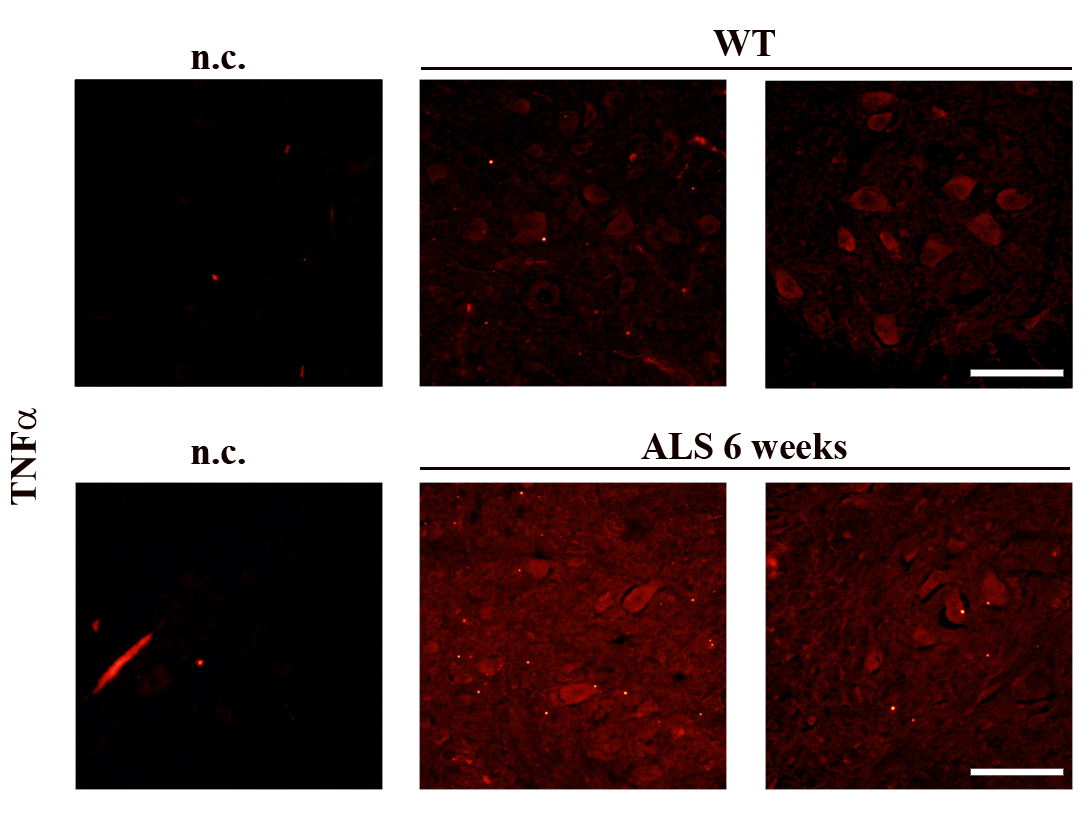

Supplement: Supplementary file 3 — Additional file 3: Figure S3. Specificity of immunofluorescence analysis of TNFα. Representative results of immunofluorescence staining of TNFα versus negative control (n.c.) in the spinal cord of WT mice and 6 weeks old SOD1G93A mice. Scale bar = 100 μm. There is a low staining of TNFα in the spinal cord of WT mice in accordance with the low level of TNFα in their lysates. [file 12974_2021_2326_MOESM3_ESM.tif]

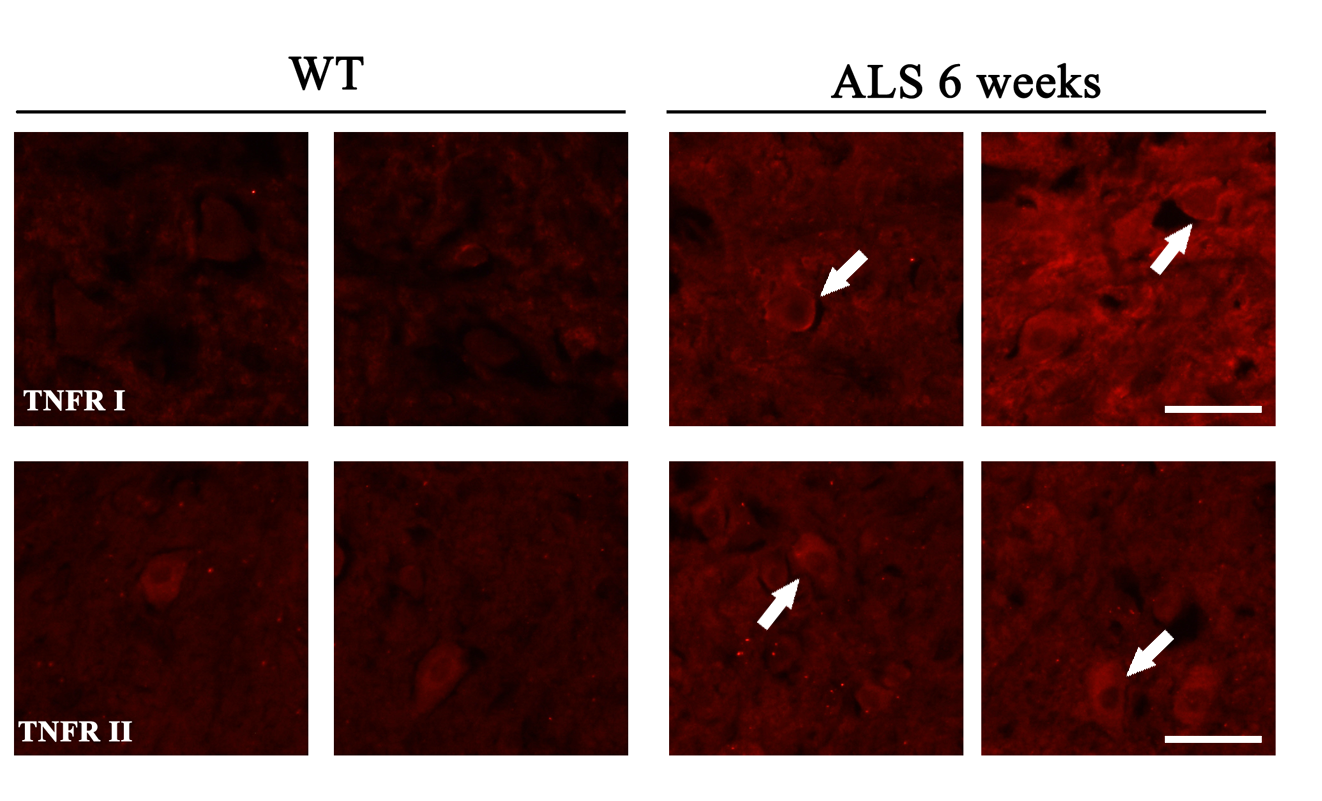

Supplement: Supplementary file 4 — Additional file 4: Figure S4. TNFα receptors are elevated in motor neurons in the spinal cord of 6 weeks old mutant SOD1G93A mice. Representative immunofluorescence staining of TNFRI and TNFRII in the spinal cord of WT mice and mutant SOD1G93A mice. Scale bars = 50 μm. The elevated receptors are detected in motor neurons as determined by the cell shape. [file 12974_2021_2326_MOESM4_ESM.tif]

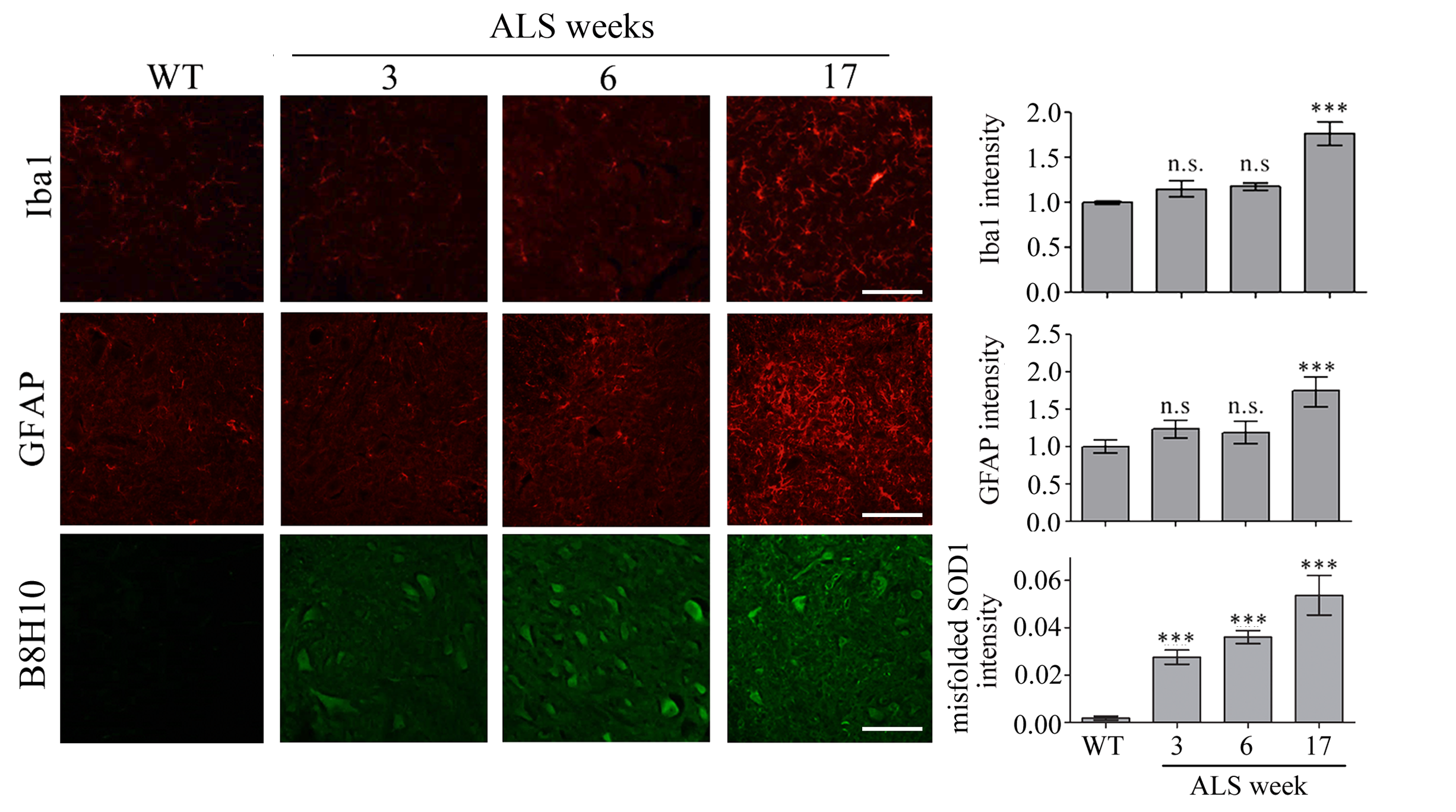

Supplement: Supplementary file 5 — Additional file 5: Figure S5. The accumulation of misfolded SOD1 precedes glia activation. Representative immunofluorescence staining of Iba1, GFAP (red) or misfolded SOD1 (B8H10, green) proteins in the lumbar spinal cord sections of WT and mutant SOD1G93A mice during the course of the disease (3, 6 and 17 weeks). Scale bars = 100 μm. The mean ± SEM fluorescence intensity expressed by arbitrary units is presented in the bar graph (n = 4 mice for each time point, five fields were analyzed for each mouse). ***p < 0.001—compared to control mice (WT). n.s. = non significant. [file 12974_2021_2326_MOESM5_ESM.tif]

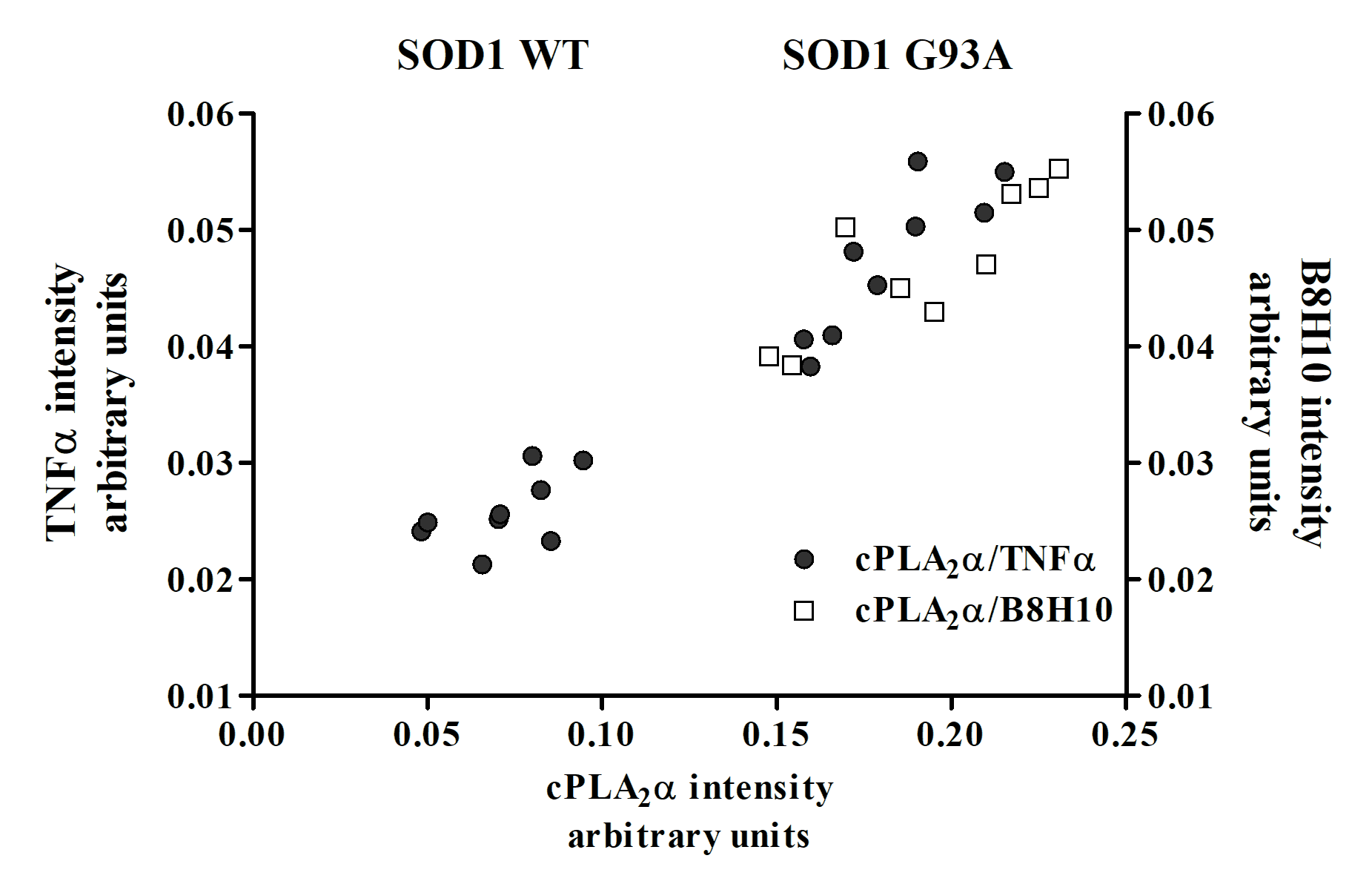

Supplement: Supplementary file 6 — Additional file 6: Figure S6. Detection of cPLA2α and TNFα in motor neurons expressing hSOD1WT or mutant hSOD1G93A. The Pearson coefficient correlation between cPLA2α and misfolded SOD1 (r = 0.87) and between cPLA2α and TNFα (r = 0.96) in motor neurons expressing mutant SOD1G93A. Representative results of florescence intensity of immunostaining presented in Figs. 3C and 6C is expressed in arbitrary units. [file 12974_2021_2326_MOESM6_ESM.tif]

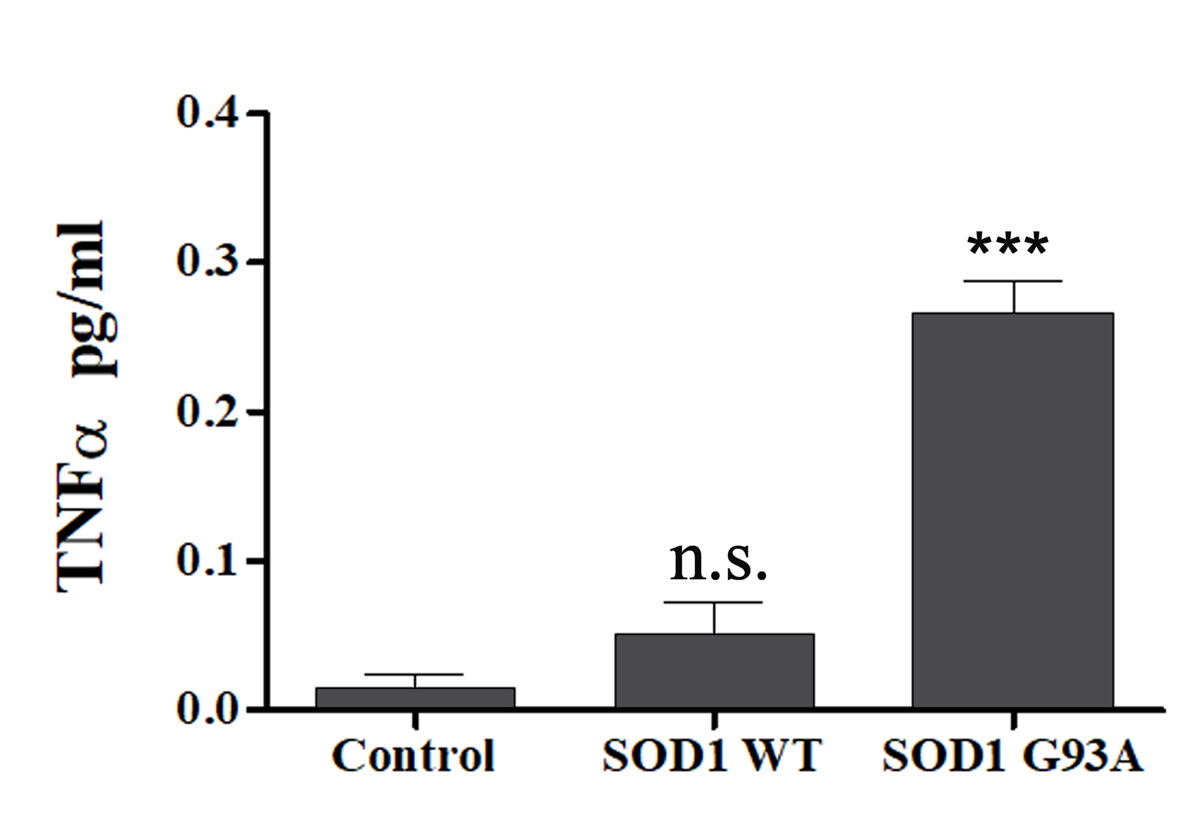

Supplement: Supplementary file 7 — Additional file 7: Figure S7. Elevated TNFα levels in supernatant of NSC34 cells expressing SOD1G93A. The levels of TNFα in the supernatants of NSC34 cells transfected with human SOD1WT or mutant SOD1G93A plasmids (as described in Fig. 6B) detected by ELISA. The bar graph is the mean ± SE of three experiments. Significance compared to control ***p < 0.001, n.s. non-significant. [file 12974_2021_2326_MOESM7_ESM.tif]
